# Impostor phenomenon in registered dietitians: an exploratory survey

**DOI:** 10.1186/s40795-023-00720-1

**Published:** 2023-05-18

**Authors:** Jennifer L. Hernandez, Nanette V. Lopez

**Affiliations:** 1 PhD Candidate in Curriculum & Instruction, Northern Arizona University, 1100 South Beaver Street, PO Box 15095, Flagstaff, AZ 86011 USA; 2 Northern Arizona University, 1100 South Beaver Street, PO Box 15095, Flagstaff, AZ 86011 USA

**Keywords:** Survey, Registered dietitian, Impostor phenomenon, Imposter syndrome, Confidence

## Abstract

**Background:**

Impostor phenomenon, also referred to as imposter syndrome or impostorism, was initially identified among high achieving women who felt undeserving of their successes because they felt they were earned through luck or chance, rather than skill or experience. It’s prevalence has been identified in many health professions; however, there are no known studies of Registered Dietitians’ (RDs) perceptions of impostor phenomenon. This study assesses the following among RDs: [1] prevalence of impostor phenomenon and differences in impostor phenomenon levels, if any, based on [2] highest educational level achieved and [3] years of experience as an RD.

**Methods:**

A cross-sectional survey was sent electronically to 5,000 RDs credentialed by the Commission on Dietetic Registration in the United States. Respondents’ agreement with 20 impostor phenomenon statements from the Clance Impostor Phenomenon Scale were measured. The sum score from the scale was used to classify levels of impostor phenomenon. Descriptive statistics and chi square analyses for comparison were evaluated.

**Results:**

Of the 445 (9%) who began the survey, 266 (5%) completed it and were included in analyses. Over 76% of 266 individuals reported at least moderate impostorism (score of 40 or fewer points out of 100). No difference was seen based on educational level (*p* = .898); however, those with less than five years’ experience reported higher impostor phenomenon (*p* < .05). Among those with five to 39 years’ experience, over 40% reported moderate impostorism.

**Conclusion:**

Impostor phenomenon is prevalent among RDs. Moderate impostorism was pervasive among all those with less than 40 years’ experience and could potentially negatively impact these respondents. Future research could explore ways to reduce impostor phenomenon in RDs.

## Introduction

In the United States, credentialed nutrition and dietetics practitioners are called Registered Dietitians or Registered Dietitian Nutritionists (RDN) [1]. There are multiple pathways into the profession, [2] and despite some technical differences, they all require didactic education of a minimum of a bachelor’s degree and a minimum of 1000 h of supervised practice [1]. As of 2021, there were 119,249 RDNs, of which 92% reported female and 80% white [3]. Among a sample of 6314 nutrition professionals, 50% reported having a master’s degree and 4% a doctorate [4].

Impostor phenomenon, also known as impostor syndrome or impostorism, has been described as a sense of not deserving the success that has been received [5]. Initially identified in women, high-achieving people who experience imposter phenomenon feel like luck, a mistake, or some sort of deception has led to the perception of success. Those experiencing it often credit external factors, such as interpersonal relationships, as opposed to internal factors, such as intelligence, for their achievements. Feelings of anxiety, depression, and low self-confidence correlate with imposter phenomenon. Research indicates that there is no difference in the prevalence of impostor phenomenon in men compared to women [6].

Imposter phenomenon has been described within various health care professions, including nurses, [7, 8] nursing students, [9, 10] physicians, [11–15] medical interns and residents, [16, 17] medical students, [18–20] pharmacy students and residents, [21, 22] and physician assistants [23]. A perspective article has been published discussed factors that may contribute to the development of impostor phenomenon in dietitians and dietetic students, including the competitive educational system, job opportunities, and the prevalence of social media, as well as strategies for addressing impostor phenomenon [24]. Despite this, no studies examining the prevalence of impostor phenomenon among Registered Dietitians (RDs) were found at the time this study was planned and conducted. Since then, only one known study has explored impostor phenomenon in this population [25].

Considering that impostor phenomenon was initially identified in highly achieving women, [5] and the profession of dietetics in the United States is predominantly women, [3] many of whom hold advanced degrees, [4] we sought to identify the following: (1) the prevalence of imposter phenomenon among RDs in the United States, (2) if imposter phenomenon differs by highest degree earned, and (3) if imposter phenomenon differs by years of working experience as an RD.

## Materials and methods

Online surveys were sent out in November 2020 to 5,000 practitioners credentialed in the United States whose email addresses were obtained from the Commission on Dietetic Registration with an email subject line of *Your help requested - Impostor Phenomenon and Dietitians*. Physical addresses were not able to be obtained with the email addresses; therefore, only online surveys were sent. One reminder email was sent to the email address of anyone who had not completed the survey. Written informed consent was obtained from participants upon agreement to begin the survey. Participants were not compensated for their participation in the survey. No identifiable information was collected in the survey, and responses were not attached to email addresses. The institutional review board at the lead author’s affiliated institution approved this study.

The Clance Impostor Phenomenon Scale [26] is a 20-question tool to identify the severity of impostorism in respondents. Items ask about respondents’ fear of failure, fear of criticism or critique, ability to repeat successes or remain successful, and sense of worth or value as compared to others. Each item is scaled from 1 to 5, with lower scores indicating less impostorism and higher scores indicating more. No items on the scale require reverse scoring. Total scores on the scale range from 20 to 100 points. Four categories of impostor phenomenon are identified: 40 or fewer points indicates few impostor characteristics, 41 to 60 is labeled as moderate impostor phenomenon experiences, 61 to 80 as frequent impostor phenomenon feelings, and over 80 as intense impostor phenomenon. Permission to use the Clance Impostor Phenomenon Scale was obtained from Dr. Clance.

Demographic data were collected from participants. Indicating that one had not worked as an RD in the past 12 months ended the survey and no further information was collected. Categories for primary employment position were taken from the 2019 Compensation and Benefits Survey of the Dietetics Profession [27]. Additional demographic information included gender identity, highest educational degree attained, and years working as a dietitian. Gender identity was categorized as male, female, nonbinary, and none of the above or prefer not to answer. Educational degree attainment was categorized as bachelor, master, or doctoral degree. Years working as a dietitian were categorized as less than 1 year, 1–2 years, 3–4 years, 5–9 years, 10–14 years, 15–19 years, 20–29 years, 30–39 years, and 40 or more years.

The Clance Impostor Phenomenon Scale and demographic questions were entered into Qualtrics® (Provo, UT) for distribution via email. Data from Qualtrics® (Provo, UT) were uploaded to IBM SPSS Statistics 26 (Armonk, NY) for analysis. Sum scores for all line items on the Clance Impostor Phenomenon Scale portion of the survey were created to evaluate total impostor phenomenon scores for participants. Additionally, impostor phenomenon categories were created based on the Clance Impostor Phenomenon scale interpretation [26]. Descriptive statistics were used to evaluate demographic data as well as overall prevalence of impostor phenomenon within each interpretive category. Chi squared analyses were calculated to evaluate differences among groups, with significance evaluated at *p* < .05.

## Results

Of the 5000 credentialed nutrition practitioners in the United States who received the survey, 445 people began it (9% response rate). Of those, 44 did not answer any questions, 52 indicated not working as a Registered Dietitian in the past 12 months, 8 did not answer any questions beyond the qualifying question, and 70 did not answer any questions beyond demographics. A total of 266 people (5%) completed the survey.

An overview of demographics, including comparison of those who completed the survey beyond demographic questions and those who did not, is provided in Table 1. There was a significant difference in gender identity between the 266 people who completed the survey (completers) and the 70 people who only responded to demographic questions (noncompleters), [χ^2^(3, N = 336) = 12.143, *p* = .007], with more people reporting male gender in the completers group. There was a significant difference in highest degree earned between noncompleters and completers, [χ^2^(3, N = 336) = 8.351, *p* = .039], with more completers having a master’s degree and more noncompleters having a bachelor’s degree. There was no significant difference in primary position or years of experience between noncompleters and completers.

**Table 1:** Distribution of Demographic Characteristics of Respondents by Survey Completion

Characteristic	Total (n = 336)	Survey Completed (n = 266)	Survey Not Completed (n = 70)
Gender identity			
*Female*	96.1%	96.6%	94.3%
*Male*	3.0%	3.4%	1.4%
*Non-binary*	0.3%	0.0%	1.4%
*None of the above/ prefer not to answer*	0.6%	0.0%	2.9%
Highest degree earned			
*Bachelor’s degree*	39.6%	36.8%	50.0%
*Master’s degree*	57.4%	60.5%	45.7%
*Doctoral degree*	2.7%	2.6%	2.9%
*No answer*	0.3%	0.0%	1.4%
Primary position			
*Clinical Nutrition – Acute Care/Inpatient*	18.8%	19.5%	15.7%
*Clinical Nutrition – Ambulatory Care*	19.6%	20.3%	17.1%
*Clinical Nutrition – Long Term Care*	11.6%	12.0%	10.0%
*Community*	11.3%	10.5%	14.3%
*Food and Nutrition Management*	6.3%	7.1%	2.9%
*Consultation and Business*	14.0%	12.0%	21.4%
*Education and Research*	6.0%	6.4%	4.3%
*Other position not listed*	12.5%	12.0%	14.3%
Years of experience			
*Less than 1 year*	4.5%	4.1%	5.7%
*1–2 years*	11.6%	11.3%	12.9%
*3–4 years*	11.9%	11.7%	12.9%
*5–9 years*	19.6%	20.3%	17.1%
*10–14 years*	11.9%	11.7%	12.9%
*15–19 years*	7.7%	7.9%	7.1%
*20–29 years*	17.0%	15.8%	21.4%
*30–39 years*	10.4%	11.7%	5.7%
*40 or more years*	5.4%	5.6%	4.3%

**Fig. 1:**
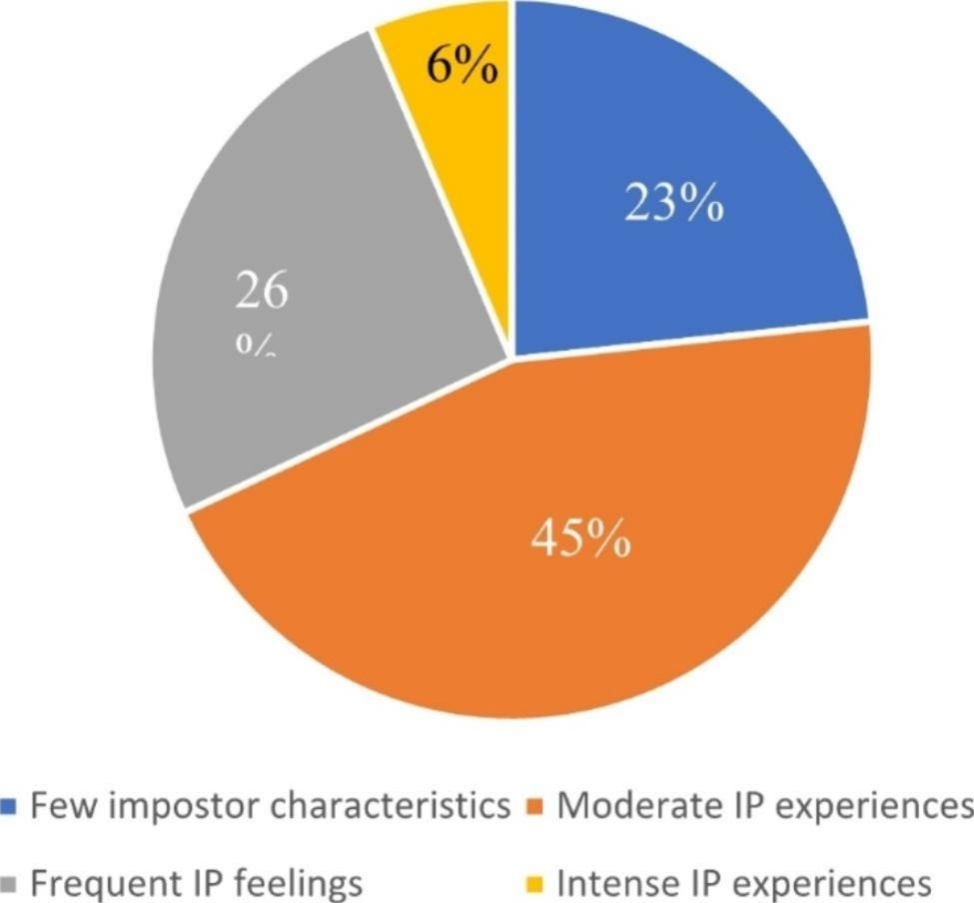
Distribution of impostor phenomenon categories among all respondents (n = 266)

**Fig. 2:**
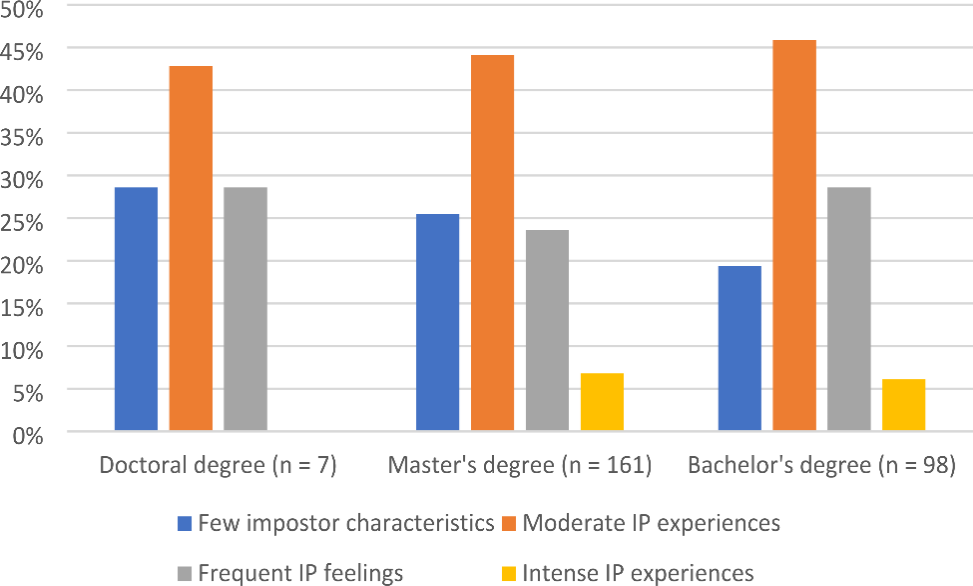
Distribution of impostor phenomenon categories by highest degree earned (n = 266)

Figure 1 presents the distribution of impostor phenomenon perception among all respondents. Figure 2 shows the percentage distribution of impostor phenomenon categories by highest degree earned. There was no significant difference in groups by degree earned, [χ^2^(6, N = 266) = 2.223, *p* = .898].

**Fig. 3:**
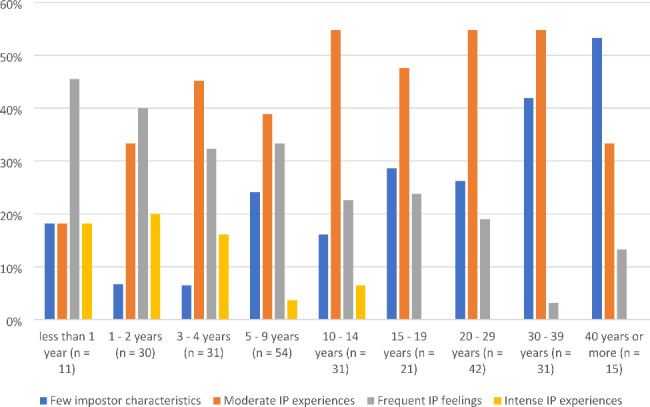
Distribution of impostor phenomenon categories by years of experience as a Registered Dietitian (n = 266)

Figure 3 provides the distribution of impostor phenomenon categories by years of experience as a Registered Dietitian. There was a significant difference in impostor phenomenon perception based on years of experience, [χ^2^(24, N = 266) = 61.824, *p* < .001]. Post hoc analyses indicated those with less than 1 year of experience perceived more impostor phenomenon than those reporting 20–29 years (*p* < .05), 30–39 years (*p* = .001), and 40 or more years of experience (*p* < .001). Practitioners with 1–2 years of experience reported more impostor phenomenon than those with 5–9 years (*p* < .05), 10–14 years (*p* < .05), 15–19 years (*p* < .05), 20–29 years (*p* < .001), 30–39 years (*p* < .001), and 40 or more years of experience (*p* < .001). Practitioners with 3–4 years of experience perceived more impostor phenomenon than those reporting 20–29 years (*p* < .05), 30–39 years (*p* < .001), and 40 or more years of experience (*p* < .001). Practitioners with 5–9 years of experience perceived more impostor phenomenon than those reporting 40 or more years of experience (*p* < .05). There were no other statistically significant differences between the groups.

## Discussion

The aims of this study were to identify (1) the prevalence of impostor phenomenon among RDs in the United States, (2) if impostor phenomenon differs by highest degree earned, and (3) if imposter phenomenon differs by years of working experience as an RD. Impostor phenomenon was identified in 76% of Registered Dietitian participants, with no significant differences in highest degree attained; however, impostor phenomenon was more prevalent in those with less than 5 years of experience in nutrition and dietetics.

Registration demographics indicate that 92% of RDs are female; [3] 96% of our sample reported female. Highest degrees earned were reported in a large-scale survey of 4692 RD participants at rates of 44% bachelor’s degree, 52% master’s degree, and 4% doctorate, [4] as compared to 36.8%, 60.5%, and 2.6% respectively in the current sample. Our sample had slightly higher proportions of women and people with master’s degrees. While somewhat similar to the overall RD population, our results are not generalizable to a broader group of dietitians.

Among our respondents, 23.3% demonstrated few impostor characteristics, with over 76% reporting at least moderate impostorism. Among those experiencing impostor feelings, 44.7% reported moderate, 25.6% frequent, and 6.4% intense impostor phenomenon. Although we cannot directly compare our results to others because no previous studies of impostor phenomenon in RDs exist, researchers have identified high levels of impostor phenomenon in other health care professionals. Among physician assistant students and recent graduates (n = 83), 50% of female respondents and 25% of male respondents reported feelings of impostorism [23]. In a pilot study of students entering a nursing program with a non-nursing bachelor’s degree (n = 27), 70% of participants were identified as having impostor phenomenon [9]. Among family medicine residents, one study (n = 194) found that 41% of women and 24% of men reported impostorism [17]. Thus, it appears that a higher proportion of dietitians may experience impostor phenomenon than new practitioners in family medicine, physician assisting, and nursing.

Because impostor phenomenon was initially identified in high achieving women [5] and 92% of nutrition professionals credentialed by the Commission in Dietetic Registration are female, [3] this study explored differences in impostor phenomenon among RDs with different highest levels of education. In the current sample, no statistically significant differences were found. Despite the conventional belief that more degrees equate to greater achievement, one could argue that anyone who successfully navigates the educational pathways to becoming a Registered Dietitian, including passing the registration examination, is a high achiever. While statistical differences of impostor phenomenon categories by highest degree earned were not identified, there are still notable findings. Among those with a bachelor’s degree as the highest educational level (n = 98), over 80% exhibited at least moderate impostor phenomenon. That number decreased to over 70% of people with a doctoral degree (n = 7). Between 40 and 50% of respondents in each educational category reported moderate impostorism. No respondents with a doctoral degree reported intense impostor phenomenon experiences. All participants reporting a doctoral degree had at least 10 years of experience in the field of nutrition and dietetics, and our results indicate that impostor phenomenon is inversely related to experience.

The third aim of this study explored differences in impostor phenomenon among those with various years of experience as a dietitian. The highest levels of impostor phenomenon were identified in people with less than 5 years of experience, with the mean impostor phenomenon score incrementally decreasing with each increase in years of experience category. In those with less than 3 years of experience, “frequent” – the third category of impostor phenomenon – had the highest frequency. Among those with 3 to 39 years of experience, moderate impostor phenomenon was most common, representing over 40% of respondents in all categories except those with 5–9 years of experience. The category of “few impostor characteristics” only represented the majority in those who reported over 40 years of experience. Another notable finding is that no one with 15 or more years of experience reported intense impostor phenomenon experiences. When initially identified as a construct, impostor phenomenon was thought to be self-perpetuating [5]. Prata and Gietzen, [23] in their study of physician assistant students and graduates, found that impostorism decreased in graduates with 3–5 years of experience, contradicting previous beliefs about the concept. Their decrease is similar to our findings of fewer impostor phenomenon with time and experience. In the book *Outliers*, Gladwell [28] identified that it takes a person 10,000 h to become an expert in an area. It is possible that new practitioners carry impostor feelings as they enter the field, and that impostorism decreases as they approach and achieve expert levels of practice. However, moderate impostorism seems pervasive as originally identified by Clance and Imes [5].

The response rate to our survey was low at only 9%, with a response rate of less than half of that of the 2019 Compensation and Benefits Survey which had a similar population [27]. Despite statistically significant differences in gender identity in our sample, gender breakdown is similar to other reports of US RDs [3, 27]. Our sample had slightly higher rates of people reporting a master’s degree (60.5%) and similar rates of doctoral degree (2.6%) as those completing the Compensation and Benefits Survey (50% and 3%, respectively) [27].

Differences between our respondents and the respondents in the Compensation and Benefits Survey were also noted in primary work position and years of experience. A much smaller percentage of our respondents reported working in acute care/ inpatient settings. A smaller percentage was also noted in those reporting working in food and nutrition management. Larger percentages of respondents in our survey reported working in ambulatory care, long-term care, community, consultation, and business. More of our respondents reported less than 1 year, 1–4 years, and 10–19 years of experience [27].

### Strengths and limitations

A novelty of this study is that it is one of the first known survey of impostor phenomenon in Registered Dietitians. Contact information for participants was obtained from a randomly selected cross-section of credentialed practitioners that was provided by the Commission on Dietetic Registration. While we cannot directly evaluate differences between people who opted to complete our survey and those who did not, we can compare our participants to those who ended the survey after completing demographics only as well as to other large-scale surveys of RDs. Additionally, the Clance Impostor Phenomenon Scale, the tool used to evaluate impostorism in this survey, has been validated in prior research [29].

This study is not without limitations. Our survey had a low response rate (9%). Considering that the survey was distributed approximately 8 months into the worldwide COVID-19 pandemic, and that many RDs were considered essential workers, they may have felt overwhelmed with work responsibilities and not filled out the survey. We implemented a cross-sectional study that evaluated impostor phenomenon at one specific point in time. Thus, we are unable to examine if impostor phenomenon decreases in individuals over time.

This survey did not collect race/ ethnicity data from respondents. It was not the aim of this study to identify differences in impostor phenomenon by race/ ethnicity. Considering the small percentage of non-white RDs, [3] it is unlikely that this cross-sectional survey would have obtained adequate participation to identify differences. A future study may seek to explore differences by race/ ethnicity. As such, the results of this study cannot be generalized to the broader RD population.

## Conclusions

Impostor phenomenon has been identified in high achieving women, [5] students and practitioners in other healthcare fields, [7–23] and now in Registered Dietitians. Impostor phenomenon has been linked to reduced self-confidence which may impact patient care and cause practitioners to feel like they constantly need to defend their position [8, 21]. RDs who battle impostor phenomenon may second guess their clinical and professional judgement. They may back down to medical staff or more senior colleagues when confronted, feeling like their opinions, decisions, and plans of care have less merit than those around them. This could lead to delayed or inappropriate care in the form of inappropriate diet orders, inappropriate nutrient content of menus, and increased risk for poor nutritional outcomes.

Despite impostor phenomenon being identified as a construct in high achieving women, no differences were seen among participants with varying degrees. All Registered Dietitians could be considered high achieving, because in order to earn the credential they successfully navigated the educational requirements, completed a competitive practical experience, and passed the Registration Examination for Dietitians [30].

Within the current study sample, people with greater years of experience reported fewer impostor phenomenon feelings. Dietetics curricula could be enhanced by alerting students to this concept, helping them to feel like they are not alone or abnormal, and reassuring that these perceptions can be managed. Future research could explore if impostor phenomenon decreases within individuals over time. Considering the large proportion of dietitians of all degree and experience levels, the field of nutrition and dietetics would greatly benefit from additional research exploring interventions for reducing impostorism in individuals.

## Data Availability

Data for the study are not publicly available and can be obtained from the corresponding author until data destruction on August 25, 2025.

## List of Abbreviations

RD: Registered Dietitian
